# Effect of Minimizing Light Exposure with Digital Visualization on Macular Function After Cataract Surgery in Patients with AMD: A Randomized Controlled Trial †

**DOI:** 10.3390/jcm15134897

**Published:** 2026-06-24

**Authors:** Otman Sandali, Rachid Tahiri Joutei Hassani, Isabelle Audo, Vincent Gualino, Christine Tchikladze Merand, Vincent Borderie

**Affiliations:** 1 Quinze-Vingts National Ophthalmology Hospital, 75012 Paris, France; isabelle.audo@inserm.fr (I.A.); vincent.borderie@upmc.fr (V.B.); 2 Service de Chirurgie Ambulatoire, Hôpital Guillaume-de-Varye, 18230 Bourges, France; 3 Department of Ambulatory Surgery, Granville Hospital, 50400 Granville, France; tjhr78@hotmail.com; 4 Department of Ophthalmology, Clinique Honoré Cave, 82000 Montauban, France; vincent.gualino@gmail.com; 5 Clinical Research Department, ELSAN, 75008 Paris, France; tchikladze-merand@elsan.care

**Keywords:** low-light exposure, cataract surgery, age-related macular degeneration, phototoxicity, macular thickness, electroretinography

## Abstract

**Purpose**: To assess whether reducing intraoperative light exposure preserves macular function after cataract surgery in patients with AMD. **Methods**: A total of 42 eyes of 42 patients with AMD were randomized in a prospective study. The primary outcome was the change in photopic (ERG) b-wave amplitude at one hour after surgery (V2). Secondary outcomes included ERG implicit time, multifocal ERG, visual acuity, and macular thickness, assessed at V2, V3 (day 1), and V4 (1 month). **Results**: Mean intraoperative light exposure was significantly lower in the 3D group than in the standard group (3938 vs 47,142 lux, *p* < 0.001). At 1 h after surgery, the decrease in photopic b-wave amplitude did not differ significantly between the two groups (−1.83 µV; 3D group, vs. −1.56 µV; standard group, *p* = 0.76). In exploratory analyses, ERG implicit time increased significantly in the standard group (*p* = 0.02) but remained stable in the 3D group (*p* = 0.24). At 1 month, an increase in macular thickness was observed only in the standard group (V1 265.9 ± 27.7 µm, V4 278.8 ± 34.9 µm; *p* = 0.003). **Conclusions**: Digital visualization significantly reduced intraoperative light exposure. However, no significant difference was observed for the primary endpoint of photopic ERG b-wave amplitude at 1 h postoperatively. Secondary findings regarding ERG implicit time and macular thickness should be considered exploratory and require confirmation in larger studies.

## 1. Introduction

Cataract surgery is frequently performed in patients with age-related macular degeneration (AMD) due to the aging of the population [1].

AMD consists of a spectrum of manifestations extending from medium-sized drusen at early stages to large drusen and pigmentary alterations at intermediate stages and to geographic atrophy or neovascularization with severe visual impairment at advanced stages [2]. Cataract surgery presents specific challenges in patients with AMD due to the potential vulnerability of the macula to photic injury during surgery, raising concerns about light-induced retinal damage that may aggravate pre-existing retinal dysfunction, even during short surgical procedures. Indeed, several cases of phototoxicity have been reported in patients with a healthy macula undergoing cataract surgery [3,4]. Patients with AMD display abnormal light adaptation and delayed recovery after light exposure [5].

A recent report by Rosenberg et al. demonstrated faster visual recovery on the first postoperative day in patients undergoing cataract surgery under low-light conditions compared with those operated on under standard microscope illumination [6].

Recent advances in digital microsurgical visualization systems have made it possible to reduce light exposure during surgery without compromising visualization quality. Several advantages of low-light surgery have been reported, including improvements in patient comfort, with less glare at the start of the procedure and faster visual rehabilitation [6,7]. A recent pilot study investigated the advantages of low-light exposure in vitrectomy for idiopathic epiretinal membranes, but objective evidence of its functional benefit in cataract surgery is still lacking [8].

Electroretinography (ERG) is a reproducible objective method for evaluating both global and localized retinal function. The amplitude of the photopic b-wave reflects cone-driven bipolar cell activity, whereas multifocal ERG provides a topographic map of macular responses. These tools are, therefore, particularly relevant for monitoring postoperative macular function [9,10].

The primary objective of this prospective study was to use electroretinography (ERG) to investigate whether cataract surgery performed under low-light 3D digital visualization preserves macular function in the early postoperative period in patients with early or intermediate age-related macular degeneration (AMD). The secondary objectives included assessments of visual acuity, anatomical macular integrity using spectral-domain optical coherence tomography (SD-OCT), and safety outcomes.

## 2. Materials and Methods

### 2.1. Study Design

This prospective, randomized, controlled, single-center study was conducted at Guillaume de Varye Clinic, Bourges, France, between 14 September 2023 and 9 August 2024. The study received approval from the South-Mediterranean II Ethics Committee (Approval No. 23.01350.000209/223 D02; approval date: 17 May 2023) and was registered at ClinicalTrials.gov (NCT05988827). This study was conducted and reported in accordance with the CONSORT guidelines. The CONSORT checklist and the study protocol are provided as Supplementary Materials. All participants provided written informed consent, and study data were anonymized prior to analysis. All methods were performed in accordance with the relevant guidelines and regulations, including the principles of the Declaration of Helsinki.

### 2.2. Study Population and Setting

This study was conducted in a specialized ophthalmology center. Eligible patients were recruited during routine clinical consultations with an ophthalmic surgeon. This series included consecutive patients over the age of 60 years with an indication for cataract surgery and a diagnosis of early or intermediate AMD. Early AMD was defined as the presence of medium-sized drusen (63–125 µm), whereas intermediate AMD was defined as the presence of large drusen (>125 µm) and/or pigmentary abnormalities.

The exclusion criteria were advanced AMD (geographic atrophy, neovascular AMD), prior ocular surgery, advanced glaucoma, diabetic retinopathy or treatment with hydroxychloroquine.

### 2.3. Randomization

Randomization was performed according to the study protocol by an independent data manager from ELSAN using R software (version 4.2.1). Patients were stratified according to cataract nuclear opalescence classification (NUC), and a computer-generated random sequence with a 1:1 allocation ratio was used. In one group, surgery was performed under 60% microscope illumination with standard visualization, and in the other, surgery was performed under 5% microscope illumination with digital visualization via the NGENUITY® 3D Visualization System (v1.4; Alcon, Fort Worth, TX, USA).

### 2.4. Surgical Technique

**Figure 1:**
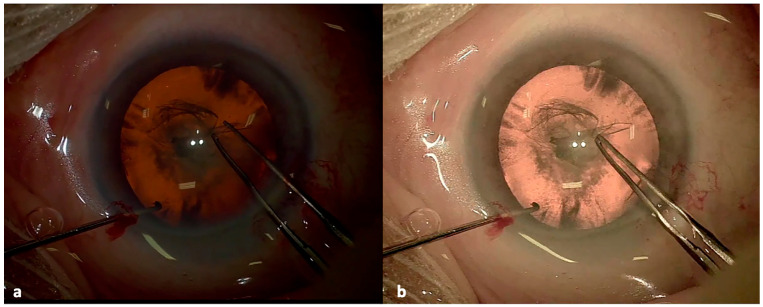
Two intraoperative images taken within a very short interval under a microscope illumination set at 5%, illustrating how digital 3D visualization enhances the brightness of the image perceived by the surgeon: (**a**) Initial image before optimization, (**b**) image after optimization with the 3D visualization system.

All operations were performed by a single experienced surgeon (OS) using a Lumera 700 (Carl Zeiss Meditec, Jena, Germany) microscope and a Centurion® Vision System (Alcon, Fort Worth, TX, USA) with the standard stop-and-chop technique. The surgeon was allowed to increase light intensity during surgery if required, and any changes in light intensity were recorded. Microscope light intensity was measured using the same calibrated luxmeter (Voltcraft MS-1300-ISO; 0.1–50,000 lx; Voltcraft, Hirschau, Germany) positioned at the focal distance under the microscope throughout the study. Measurements were systematically recorded at the beginning of each procedure and whenever the surgeon adjusted the illumination settings. Total intraoperative light exposure was subsequently calculated in lux·min for each procedure. For the NGENUITY® 3D Visualization System, the gain was set to 2 and the diaphragm aperture was set to 80%. Brightness was decreased to 44 (47.8 for the default setting) and gamma was increased to 1.4 (1.20 for the default setting). Color saturation was decreased to 30% (90% for the default setting). Figure 1 illustrates how digital settings can artificially increase the perceived light in the image.

### 2.5. Outcome Measures

Several parameters were recorded, including intraoperative parameters such as cumulative dissipated energy (CDE), surgery duration and light exposure during surgery, and clinical parameters, such as best corrected visual acuity (BCVA) expressed as logMAR, corneal and macular thicknesses, focal ERG, and multifocal ERG (mERG). Examinations were performed before surgery (V1), one hour after surgery (V2), the day after surgery (V3), and one month after surgery (V4). ERG acquisition and analysis were performed by examiners who were masked to treatment allocation throughout the study. The primary outcome was the change in photopic ERG b-wave amplitude, measured in µV, from V1 to V2 (i.e., one hour after cataract surgery relative to baseline), between patients operated with NGenuity and those operated under standard ocular microscope (SOM) light conditions.

Secondary outcomes included BCVA and b-wave latency time measured at V1, V2, V3, and V4, as well as multifocal ERG (mfERG) and macular thickness measured at V1, V3, and V4.

### 2.6. Sample Size Calculation

Patients were enrolled under the following hypotheses: expected difference in the primary endpoint between the two groups = 20%, alpha risk = 5%, power = 90%, expected standard deviation = 3.75. Based on these values and including 15% of dropouts, we calculated that 21 patients would be required for each group, giving a total of 42 patients.

### 2.7. Statistical Analysis

Continuous variables are expressed as means ± standard deviations, whereas categorical variables are expressed as frequencies and percentages. The normality of the data distribution was assessed with the Shapiro–Wilk test. Intrapatient comparisons between examinations were performed with paired Student’s *t*-tests for normally distributed data or Wilcoxon signed-rank tests for data not following a normal distribution. This statistical method was used for the main outcome (difference: V2-V1). For quantitative secondary outcomes, repeated-measures ANOVA including group as fixed factor, time (for three or more time points) as a repeated factor, baseline as a covariate, and the grouptime interaction term, or Friedman tests, were used for variables assessed across multiple time points. Interpretation of the results was based on the group time interaction and only these comparative results are presented in this manuscript. When a time effect or an interaction effect was present, multiple comparisons were performed by the statistical model. All statistical tests were two-tailed, and *p*-values < 0.05 were considered statistically significant. Analyses were performed with R (version 4.4.2).

## 3. Results

Demographic and baseline characteristics: Seven of the 42 patients initially enrolled were excluded from the final analysis. 

**Figure 2:**
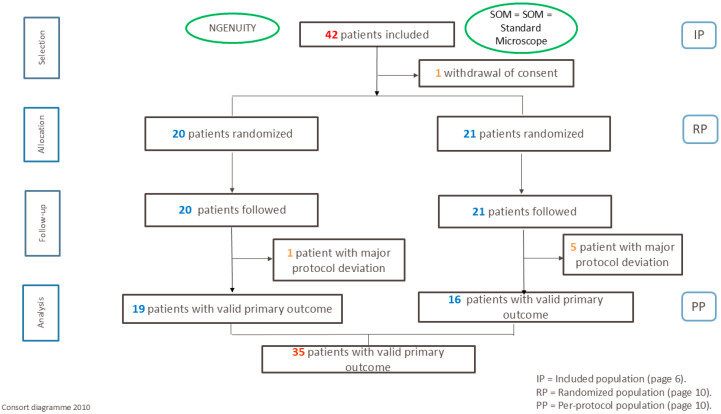
Flow diagram of the study population showing patient inclusion, randomization, follow-up, and analysis.

One patient withdrew consent before surgery, and six patients were excluded because the primary endpoint could not be reliably assessed at V1 and/or V2 due to major ERG acquisition issues, including uninterpretable recordings, electrode placement errors, and excessive recording noise. All exclusions were determined prior to statistical analysis. The final cohort included 35 patients: 19 patients who underwent surgery with the Ngenuity® 3D Visualization System under low-light conditions, and 16 patients who underwent standard surgery with conventional microscope illumination (see Figure 2).

Baseline characteristics, including age, gender distribution, cataract stage according to the LOCS III nuclear opalescence classification [11], best corrected visual acuity (BCVA), ERG b-wave amplitude and latency time, and central foveal thickness, were comparable between the Ngenuity and standard groups (all *p* > 0.1) (Table 1).

Intraoperative data: The mean duration of surgery was similar in the two groups, with a mean of 9.55 ± 2.70 min for the Ngenuity group and 9.76 ± 3.18 min for the standard group (*p* = 0.59). No intraoperative complications were reported in either group.

Total intraoperative light exposure was significantly lower in the Ngenuity group (3938.3 ± 4208.0 lux·min) than in the standard group (47,141.9 ± 18,468.2 lux·min) (*p* < 0.001). The mean cumulative dissipated energy (CDE) was 8.46 ± 2.53 in the Ngenuity group and 7.97 ± 2.53 in the standard group (*p* = 0.75).

Visual acuity outcomes: All patients presented a transient decrease in visual acuity one hour after surgery, followed by a progressive improvement on day 1 and at one month. No statistically significant differences in BCVA were observed between the NGENUITY and standard groups at any postoperative time point (all *p* > 0.1). Mean BCVA values in the NGENUITY and standard groups were 0.877 ± 0.179 and 0.925 ± 0.133 logMAR at V2, 0.169 ± 0.154 and 0.153 ± 0.137 logMAR at V3, and 0.031 ± 0.060 and 0.040 ± 0.093 logMAR at V4, respectively.

Focal electroretinography (ERG): Mean photopic b-wave amplitude for the total population decreased significantly from 12.15 ± 4.42 µV at baseline (V1) to 10.10 ± 4.45 µV one hour after surgery (V2, *p* < 0.001), and remained significantly lower than the baseline value on day 1 (V3: 10.08 ± 3.64 µV, *p* < 0.001 vs. V1). By one month (V4), amplitude values had returned to levels similar to those at baseline (V1 vs. V4, *p* = 0.31). Regarding the primary endpoint (change in ERG b-wave amplitude, from V1 to V2), no significant difference was found between the two groups: −1.83 ± 1.96 µV in Ngenuity group and −1.56 ± 2.72 µV in standard group (*p* = 0.76). No significant changes were observed between the two groups at V3 and V4 (*p* = 0.52).

Implicit b-wave time: In the standard group, implicit time increased significantly from baseline to V2 (V1: 37.59 ± 1.89 ms; V2: 38.81 ± 2.31 ms; *p* = 0.02) and remained stable in the Ngenuity group (V1: 37.21 ± 1.31 ms; V2: 37.26 ± 1.56 ms; *p* = 0.24). 

Multifocal ERG: No statistically significant differences in any multifocal ERG parameters were found between the Ngenuity and standard groups in assessments based on absolute amplitude or amplitude ratios across rings 1 through 5 (*p* > 0.1) (Table 2).

OCT macular thickness: Mean central macular thickness at baseline was 263.2 ± 25.3 µm for the total cohort. It remained stable on the day after surgery (V3), at 260.2 ± 22.4 µm, and then increased slightly but significantly to 274.7 ± 40.6 µm at one month (V4) (*p* < 0.05). An exploratory subgroup analysis revealed that this increase was significant between baseline and one month in the standard surgery group (V1 265.9 ± 27.7 µm, V4 278.8 ± 34.9 µm *p* = 0.003). In contrast, in the group undergoing surgery under low-light conditions, this increase was not significant (V1 260.3 ± 23.6 µm, V4 270.4 ± 47.3 µm *p* = 0.13).

No serious postoperative complications occurred in either group. Two patients in the Ngenuity group developed transient macular edema at V4, with full resolution after one month of follow-up with anti-inflammatory eye drops. Compliance with early postoperative treatment was poor in one patient and the other had a thin epiretinal membrane before surgery.

## 4. Discussion

This prospective study evaluated the impact on early functional and anatomical retinal changes after cataract surgery of minimizing intraoperative light exposure in patients with age-related macular degeneration (AMD). Two comparable groups were studied under two different lighting conditions. The change in photopic b-wave amplitude one hour after surgery did not differ significantly between groups. Exploratory analyses showed a significant increase in b-wave latency in the standard group, whereas no significant change was observed in the Ngenuity group. At one month, a significant increase in central macular thickness was observed only in the standard group.

Cataract procedures are short, but high levels of light exposure during such procedures may present a potential risk in patients with macular disease [3,4]. The transient decrease in b-wave amplitude and increase in implicit time observed one hour and one day after surgery are consistent with previous reports describing transient postoperative alterations in retinal electrophysiological function [12]. Implicit time remained stable in the Ngenuity group at V2, whereas a significant increase was observed in the standard group, suggesting a possible difference in early postoperative retinal functional changes between groups. By one month, b-wave amplitudes had returned to baseline, indicating a reassuring recovery of retinal function in these patients with AMD.

Visual acuity improved progressively after surgery in all patients, with no significant differences between groups. Contrary to the results of Rosenberg et al., who reported rapid visual recovery on the first day after surgery in patients undergoing procedures in low-light conditions, we found no such difference in this study, possibly because of the smaller sample size of our cohort [6].

Central macular thickness increased slightly only in the standard group at one month after surgery, raising the hypothesis that higher intraoperative light exposure may contribute to subtle macular thickening, potentially through low-grade photic or inflammatory stress [13,14,15,16]. Our findings are consistent with those of Rosenberg et al., who reported that high levels of light exposure during cataract surgery are associated with postoperative macular thickening, supporting the hypothesis that light may be one of multiple factors contributing to the pathogenesis of postoperative macular edema [6]. It should be noted, however, that the differences observed for ERG implicit time and central macular thickness were modest in magnitude, and their clinical relevance remains uncertain. These findings should therefore be interpreted with caution and considered exploratory, particularly in the context of multiple secondary outcomes.

Digital imaging allows artificial enhancement of the luminance perceived by the surgeon on the 3D screen without decreasing image resolution. In this study, this technology allowed the surgeon to operate safely without increasing the amount of microscope light reaching the patient, which was set to 5% in all cases. The use of 3D visualization technology was introduced in ophthalmology in 2008 and has since been shown to be of considerable clinical value due to its ability to improve resolution and depth of field, as well as its ability to apply color filters during surgery [17,18,19,20]. For surgeons who do not have access to this technology, several practical tips can help to reduce the amount of light delivered during surgery significantly. First, the microscope light can be switched off during surgical downtime, such as when changing gloves, preparing the intraocular lens, or while waiting for the nurse to retrieve instruments outside the operating room. Intraoperative light exposure can be significantly reduced during the initial steps preceding phacoemulsification, and during the final stages, such as cortex removal and intraocular lens implantation, without compromising visualization, due to the high-quality retroillumination available at these stages. It has also been suggested that tilting the microscope slightly to shift the light beam can prevent direct macular exposure [21].

The main limitation of this study was its small sample size, which may have reduced the statistical power for detecting subtle differences between groups. In addition, the follow-up period was limited to one month and therefore does not allow assessment of longer-term functional and anatomical outcomes in patients with AMD. Furthermore, the assumptions used for the sample size calculation were not confirmed by the observed data. The results of the present study may help refine these assumptions and facilitate the design of future studies with larger sample sizes and longer follow-up periods.

Another limitation is that the 1 h postoperative ERG assessment was specifically selected to investigate potential very early retinal functional changes; however, findings obtained at this early postoperative time point should be interpreted with caution due to the potential influence of early perioperative factors. Finally, although statistically significant differences were observed for ERG implicit time and central macular thickness, these changes were modest in magnitude and their clinical relevance remains uncertain.

In conclusion, low-light digital visualization significantly reduced intraoperative light exposure during cataract surgery in patients with early or intermediate AMD. However, no significant difference was observed for the primary endpoint of photopic ERG b-wave amplitude at 1 h postoperatively. The observed differences in ERG implicit time and macular thickness should be considered exploratory and interpreted with caution. Additional studies are warranted to further investigate the potential functional and anatomical effects of reduced intraoperative light exposure on the macula in patients with AMD.

## Figures and Tables

**Table 1:** Baseline demographic and ocular characteristics of the study population.

Characteristics	Ngenuity Group (n = 19)	Standard Group (n = 16)	*p*-Value
Gender:			0.72
Male	5 (26.32%)	6 (37.5%)	
Female	14 (73.68%)	10 (62.5%)	
Cataract stage (LOCS):			0.46
2	19 (100.0%)	15 (93.75%)	
3	0 (0.0%)	1 (6.25%)	
BCVA (logMAR), mean ± SD:	0.244 ± 0.174	0.239 ± 0.156	0.96
B-wave amplitude (µV), mean ± SD:	12.44 ± 4.20	11.81 ± 4.91	0.69
B-wave Latency time (ms), mean ± SD:	37.22 ± 1.48	37.87 ± 1.90	0.25
Central foveal thickness (µm), mean ± SD:	258.9 ± 23.2	263.9 ± 25.0	0.38

**Table 2:** Multifocal ERG amplitudes (nV/deg^2^) in the Ngenuity and standard groups across concentric retinal rings at (V1), (V3), and (V4).

	Ring 1 (<2°)	Ring 2 (2°–5°)	Ring 3 (5°–10°)	Ring 4 (10°–15°)	Ring 5 (>15°)
V1					
Ngenuity	1745.9 ± 452.1	982.3 ± 180.7	915.2 ± 150.0	939.4 ± 155.8	1032.5 ± 193.6
Standard	2011.5 ± 667.0	1094.4 ± 197.8	1046.0 ± 188.7	1073.4 ± 195.2	1082.8 ± 225.5
V3					
Ngenuity	1804.3 ± 703.8	899.8 ± 231.7	853.1 ± 165.2	853.9 ± 165.2	914.5 ± 187.0
Standard	2003.4 ± 664.2	1082.4 ± 135.7	958.7 ± 142.8	966.1 ± 181.4	974.1 ± 158.8
V4					
Ngenuity	1901.8 ± 681.6	1094.3 ± 331.9	944 ± 271.5	969.8 ± 236.6	1048.2 ± 300.2
Standard (N = 14)	1827.9 ± 660.6	1064.21 ± 189.01	983.5 ± 203.1	927.4 ± 305.4	1050.8 ± 242.1
*p* value (group-time interaction)	0.37	0.17	0.97	0.37	0.95

## Data Availability

The datasets analyzed in this study are available from the corresponding author on reasonable request.

## Supplementary Materials

The following supporting information can be downloaded at: https://www.mdpi.com/article/10.3390/jcm15134897/s1, CONSORT 2010 checklist and study protocol.
