# Supplementary material for: Effect of Minimizing Light Exposure with Digital Visualization on Macular Function After Cataract Surgery in Patients with AMD: A Randomized Controlled Trial
Source: J Clin Med. 2026 Jun 24;15(13):4897. doi: 10.3390/jcm15134897 (PMC13361183; doi:10.3390/jcm15134897)
Supplement: Supplementary file 1 [file jcm-15-04897-s001.zip › Study protocol.pdf]

Effect of minimizing light exposure intensity of 3D digital visualization (NGenuity) on macular function after cataract surgery in patients with early or intermediate AMD: A comparative ERG

EMERGING

Clinical Research Protocol
Version n°1.2 of 05/15/2023

Numéro ID-RCB / EudraCT : 2023-A00815-40

Sponsor
Groupement de Coopération Sanitaire ELSAN
58 bis rue de la Boétie
75008 Paris

Principal Investigator:
Dr Otman SANDALI
Hôpital Privé Guillaume de Varye
210 route de Vouzeron
18230 Saint-Doulchard

Document History

Protocol Version	Date	Details of modifications
1.0	04/04/2023	Initial submission
1.1	04/24/2023	Comments ANSM (ISO14155)
1.2	05/15/2023	Comment CPP (Protected participant)

Signature page of protocol

« Effect of minimizing light exposure intensity of 3D digital visualization (NGenuity) on macular function after cataract surgery in patients with early or intermediate AMD: A comparative ERG»

Effet de la réduction de l'intensité lumineuse avec le système de visualisation 3D (NGenuity) durant la chirurgie de la cataracte sur la fonction maculaire des patients présentant une DMLA précoce ou intermédiaire : ERG comparative

EMERGING

ID-RCB: 2023-A00815-40

Sponsor
Groupement de Coopération Sanitaire ELSAN
58 Bis rue de la Boétie
75008 PARIS
Tél : 06 23 63 76 33
Dr Shahnaz Klouche
Email : klouche@elsan.care

Principal Investigator
Dr Otman SANDALI
Hôpital Privé Guillaume de Varye
210 route de Vouzeron
18230 Saint-Doulchard
Tel : 06 50 20 70 62
Courriel : sanotman1@yahoo.fr

General Information

**Sponsor**<br>Groupement de Coopération Sanitaire ELSAN<br>58 Bis rue de la Boétie<br>75008 PARIS<br>Tél : 06 23 63 76 33<br>Dr Shahnaz Klouche<br>Courriel : klouche@elsan.care	**Methodology and Data management**<br>Direction de la recherche du groupe ELSAN<br>58 bis rue de la Boétie,<br>75008 Paris<br><br>**Methodology** :<br>Mme Aurélia CASSANY<br>Tél. : 06 38 22 94 11<br>Courriel : cassany@elsan.care<br><br>Mme Christine TCHIKLADZE<br>Tél. : 07 85 88 77 89<br>Courriel : tchikladze-merand@elsan.care<br><br>**Data manager :**<br>Mme Sophia Machioudi<br>Tél.: 06 76 26 12 10<br>Courriel : machioudi@elsan.care
**Leading Principal investigator**<br><br>Dr Otman SANDALI<br>Hôpital Privé Guillaume de Varye<br>210 route de Vouzeron<br>18230 Saint-Doulchard<br>Tel : 06 50 20 70 62<br>Courriel: sanotman1@yahoo.fr	

Table of Contents

1. SYNOPSIS 9

2. INTRODUCTION 13

2.1. Background and scientific context 13

2.1.1. Pathology 13

2.1.2. Procedure of study 13

2.2. Study hypothesis and expected results 13

2.3. Study design rationale 14

2.4. Benefit / risk balance 14

2.5. Expected benefits 15

3. STUDY OBJECTIVES 16

3.1. Primary objective 16

3.2. Secondary objectives 16

4. ENDPOINTS 16

4.1. Primary endpoint 16

4.2. Secondary endpoints 16

5. DESIGN OF CLINICAL INVESTIGATION 16

5.1. Overall design 16

5.2. Measures to minimize biais 16

5.2.1. Randomization 16

5.2.2. Masking procedure 16

5.2.2.1. Single-masked 16

5.2.2.2. Unmasking 18

6. STUDY POPULATION 19

6.1. Inclusion criteria 19

6.2. Exclusion criteria 19

6.3. Enrollment modalities 20

7. STUDY DEVICE 20

7.1. Study device: Ngenuity 20

7.2. Comparator device: som 20

7.3. Medical device availability 20

8. TREATMENT AND PROCEDURE ASSOCIATED 20

8.1. Procedure associated: ERG 20

8.2. Authorized concomitant medications 21

8.3. Prohibited concomitant medications/procedures 21

9. STUDY PROCEDURES 22

9.1. Study duration 22

9.2. Scheduled of assessment 22

9.3. Screening visit 22

9.4. Visit 2 23

9.5. Follow-up visit 23

9.6. Last visit 23

9.7. DISCONTINUATION FROM THE STUDY 23
9.7.1. Withdrawal 23
9.7.2. Early termination 24
9.7.3. Eligibility criteria violations 24
9.7.4. Protocol deviations 24
9.8. CONSTRAINS RELATED TO THE CLINICAL INVESTIGATION 24
10. ADVERSES EVENTS 25
10.1. DÉFINITIONS 25
10.2. EVALUATION OF RELATIONSHIP 26
10.3. EVALUATION OF THE INTENSITY 26
10.4. NOTIFICATION BY THE INVESTIGATOR TO THE MATERIOVIGILANCE 27
10.5. NOTIFICATION BY THE INVESTIGATOR TO THE SPONSOR 27
10.6. NOTIFICATION BY THE SPONSOR TO AUTORITIES 27
10.7. TIMELINE AND REPORTING MODALITIES BY THE SPONSOR TO AUTORITIES 27
10.8. CLINICAL INVESTIGATION REPORT 28
11. DATA MANAGEMENT 28
12. STATISTIC METHODS 29
12.1. SAMPLE SIZE CALCULATION 29
12.2. POPULATION ANALYSIS 29
12.3. STATISTIC ANALYSIS 30
12.3.1. Overall considerations 30
12.3.1.1. Statistic Software 30
12.3.1.2. Missing data management 30
12.3.1.3. Descriptive statistics 30
12.3.1.4. Statistical tests 30
12.3.2. Description of population 30
12.3.3. Analysis of the main objective base on principal endpoint 31
12.3.4. Analysis of secondary objectives based on secondary endpoints 31
12.3.4.1. Retinal phototoxicity from V1 to V4 31
12.3.4.2. Cone response on from V1 to V4 32
12.3.4.3. Other damage from V1 to V4 32
12.3.4.4. Visual acuity From V1 to V4 33
12.3.5. Interim analysis 33
13. CLINICAL INVESTIGATION OVERSIGHT 33
13.1. COMMITTEE 33
13.2. QUALITY CONTROL 33
13.3. AUDIT AND INSPECTION 33
14. ACCESS TO DATA AND SOURCE DOCUMENTS 34
14.1. ACCESS TO DATA 34
14.2. SOURCE DATA 34
14.3. CONFIDENTIALITY 34
14.4. PERSONAL DATA PROTECTION 34
15. ETHICAL AND REGULATORY CONSIDERATIONS 35
15.1. ETHICAL CONSIDERATIONS 35
15.2. REGULATORY CLASSIFICATION 35
15.3. REGULATORY REQUIREMENTS 35

16. RIGHTS OF PATIENT **36**
16.1. INFORMATION OF PATIENTS 36
16.2. CONSENT 36
16.3. RIGHTS OF PATIENTS AND DATA ACCESS 36
17. RETENTION OF RECORDS **37**
18. FINAL REPORT **37**
19. RULES RELATED TO PUBLICATION **38**
19.1. SCIENTIFIC COMMUNICATION 38
19.2. COMMUNICATION OF RESULTS TO PATIENTS 38
20. REFERENCES **38**
Budget 39

ABREVIATIONS LIST

3D: Three-dimensional
AE: Adverse Event
AMD: Aged Macular Degeneration
ANSM: French Agency for safety of drugs and health products
CNIL : Commission Nationale de l'Informatique et des Libertés
CRF: Case report form
DPO: Data Protection Officer
e-CRF: Electronic case report form
ERG: Electroretinogram
EU: European Union
fERG: Focal ERG
GCP: Good Clinical Practices
GDPR: General Data Protection Regulation
ICH: International Council for Harmonisation of Technical Requirements for Pharmaceuticals for Human Use
IOL: Intraocular lens
ISCEV: Internal Society for Clinical Electrophysiology of Vision
MDR: Regulation (EU) 2017/745 of the European parliament and of the council of 5 April 2017 on medical devices, amending Directive 2001/83/EC, Regulation (EC) No 178/2002 and Regulation (EC) No 1223/2009 and repealing Council Directives 90/385/EEC and 93/42/EEC.
NUC: Nuclear Cataract
PBD: Prevention of Blindness and Deafness
SAE: Severe Adverse Event
SD: Standard deviation
SOM: Standard Operating Microscope
WHO: World Health Organization

1. Synopsis

**SPONSOR**	Groupement de Coopération Sanitaire ELSAN
**PRINCIPAL INVESTIGATOR**	Dr Otman SANDALI<br>Hôpital Privé Guillaume de Varye<br>210 route de Vouzeron<br>18230 Saint-Doulchard
**TITLE**	Effect of minimizing light exposure intensity of 3D digital visualization (NGenuity) on macular function after cataract surgery in patients with early or intermediate AMD: A comparative ERG»<br><br>EMERGING
**SCIENTIFIC BACKGROUND**	Cataract surgery is one of the most frequently performed surgical interventions worldwide. The development of phacoemulsification technology, instrumentation and surgical techniques has improved both the efficacy and safety of procedures, reducing rates of serious postoperative adverse events.<br>The microscope light-induced retinal toxicity after cataract surgery has been described in several reports even in short procedures; however, this potential toxicity has not been evaluated by objective criteria. Indeed, this retinal phototoxicity would be increased for patients with mild macular diseases such as early stages of AMD (Aged Macular Degeneration) (ie: drusen) which are frequently associated in elderly patients with cataract. A systematic literature review of 129 664 individuals estimated the prevalence of early macular degeneration to 8.01% in population aged 45 to 85 years.<br><br>A three-dimensional (3D) heads-up system (NGenuity®, Alcon, Fort Worth, TX) was recently introduced for both vitreoretinal and cataract surgery, radically improving the quality of surgical visualization. This system allows performing cataract surgery at a lower fraction of light intensity reducing patient's photophobia complaints during surgery and probably preventing potential phototoxic macular injury in cataract surgery.<br><br>In a recent pilot study, the Ngenuity system was reported to decrease significantly the operative light intensity and to contribute to a faster visual recovery relative to traditional microscopes in series of 35 patients (51 eyes) who underwent cataract surgery.<br>The aim of the study will be to assess the potential functional macular effects by focal and multifocal ERG after cataract

	surgery with NGenuity by comparison to Standard Operating Microscope (SOM). This study would particularly address eyes at risk for macular toxicity like patients :<br>- with early AMD: medium-sized drusen (between 63µm and 125µm in diameter) without pigmentary abnormalities<br>- with intermediate AMD : large drusen (>125 µm) or pigmentary abnormalities in the retina associated with at least medium drusen.
**OBJECTIVES**	The main objective is to assess the change from baseline of retinal response amplitude in voltage in patients one hour after cataract surgery performed with NGenuity compared to standard light condition with SOM.<br><br>The secondary objectives are to assess the change from baseline in both arms of:<br>- amplitude of the response in voltage in patients one day and one month after cataract surgery<br>- latency of response in time in patients one hour, one day and one month after cataract surgery<br>- total photoreceptors response in the macula one day and one month after cataract surgery<br>- visual acuity one hour, one day and one month after cataract surgery.
**STUDY DESIGN**	Prospective, randomized, 2-arm controlled, parallel, single-masked, single-center study.
**REGULATORY**	MDR 2017/745 (4.2)
**INCLUSION CRITERIA**	- Patient undergoing cataract surgery and presenting signs of early AMD with medium-sized drusen (between 63 µm and 125 µm in diameter) or intermediate AMD with large drusen (>125 µm) or pigmentary abnormalities with at least medium sized drusen in the study eye.<br>- Patient affiliated to health care system<br>- Patient with signed informed consent
**EXCLUSION CRITERIA**	- Children under 18 years old<br>- AMD defined by geographic atrophy or pigment epithelium detachment, subretinal hemorrhage or visible subretinal new vessel, or subretinal fibrous scars in the study eye<br>- Non-age-related cataracts<br>- History of previous ocular surgery, anterior segment (corneal, anterior chamber, sulcus) or posterior segment (uveal, vitreo-retinal) pathology including retinal vascular occlusive disease, retinal detachment or peripheral retinal laser photocoagulation, neovascular AMD, glaucoma in the study eye<br>- Any current anterior or posterior segment inflammation of any etiology, and/or history of any disease producing an intraocular inflammatory reaction.

	- Patient with conditions that increase the risk of zonular rupture during cataract extraction procedure that may affect the postoperative centration or tilt of the IOL<br>- Patient treated by plaquenil<br>- Patient already enrolled in the same study for one eye<br>- Patient already enrolled in clinical trial or with exclusion period in progress<br>- Patient under guardianship, curatorship or other legal protection, deprived of liberty by judicial or administrative decision<br>- Pregnant or breastfeeding women<br>- Patient hospitalized without consent
**PROCEDURE OF THE RESEARCH**	The procedure of the research is to perform cataract surgery under microscope using the NGenuity 3D system (CE marked, from Alcon) which consists of a 3D stereoscopic, high-definition digital video camera and workstation to provide magnified stereoscopic images of objects during micro-surgery.<br><br>Two arms of the procedure are:<br>-study device called NGENUITY: NGenuity allowing a reduced light intensity to 15%.<br>-comparator device called SOM: microscope without NGenuity with light intensity of 60%, similar to a Standad Operating Microscope (SOM).
**ENDPOINTS**	The primary endpoint is the change from baseline (V1) of the photopic b-wave amplitude (µV) measured at V2 (one hour after the cataract surgery) by fERG.<br><br>The secondary endpoints are the change from baseline (V1) of:<br>- Photopic b-wave amplitude (µV) measured at V3 (post-operative day) and V4 (1 month after surgery) by fERG.<br>- fERG implicit time (msec) measured at V2; V3 and V4<br>- Multifocal ERG amplitude (µV) measured at V3 and V4.<br>- Visual acuity measured at V2; V3 and V4 (10 scale)
**SAMPLE SIZE**	Under the following hypotheses: Superiority study, Main evaluation criterion = Photopic b-wave amplitude, Continuous parameter, 2 parallel groups, Alpha = 5%, Power = 90%, Two-sided situation, Standard deviation = 3.75, Expected difference from baseline on photopic b-wave for studied group = 20% and for standard group = 0%. If we consider a **15%** drop out (loss of FU, ERG impossible to realize at each visit, etc …): a total of **42 patients: 21 patients/arm** would be included.
**NUMBER OF SITE**	1
**CALENDAR**	- Inclusion duration: 6 months<br>- Individual duration: max 2 months<br>- Total research duration: 8 months
**STATISTICAL ANALYSIS**	Descriptive statistics according to the parameter type :<br>- Quantitative data: number of missing data, sample size, mean, standard deviation, median, first and third quartiles, minimum and maximum

	- Qualitative data: number of missing data, sample size, percentage of each item and in total. The percentage will be calculated regardless of missing data.<br>Group comparability at inclusion: Student's test or Chi² test or equivalent<br>Main and secondary criteria statistical analysis :<br>- Descriptive statistics at each evaluation time by technic groups and in total and of the difference from baseline (V1) by technic groups and in total<br>- Analysis of covariance including the following factors: Fixed factor (Technic group), Repeated factor (Time), Interaction time*group and Covariate (baseline value).<br>- Graph of change for each parameter analysed
**EXPECTED RESULTS**	Although NGenuity 3D visualization system exist since many years, there is still a lack of objective data showing less retinal phototoxicity with NGenuity by comparison to SOM (standard operating microscope) after cataract surgery. The results of the study (if primary endpoint is achieved) will benefit to the patient, and particularly to patient suffering from early AMD because it will demonstrate that a less phototoxic procedure does exist with less intense light during the cataract surgical procedure.

2. Introduction

2.1. BACKGROUND AND SCIENTIFIC CONTEXT

2.1.1. PATHOLOGY

Cataract is an opacification of the eye's lens located behind the iris leading loss of vision, until blindness if it not treated. It is a natural ageing of the lens characterized by a decrease of visual acuity, far and near vision, with altered colour vision, contrast altered and frequent glares.

Cataract condition affects more than half of the people over 65, and more than two-thirds after 75 years old, regardless of the gender. Even if age is the main risk factor, smoking, diabetes with uncontrolled glycaemia, corticosteroids, high myopia, ultraviolet radiation or other eye injuries are involved in cataract development (Brown et al, 1987; Robman et al, 2005). To date, cataract is not preventable and no cure exists; the only treatment is surgery, allowing recovery of the sight.

2.1.2. PROCEDURE OF STUDY

Cataract surgery is one of the most frequently performed surgical interventions worldwide (Foster et al, 2000). In France, more than 750 000 patients underwent cataract surgery each year. Cataract surgery is a standardized and painless procedure where the surgeon removes the eye's cloudy lens while retaining its envelope in which an artificial intraocular lens (IOL) is inserted. The development of phacoemulsification technology, instrumentation and surgical techniques has improved both the efficacy and safety of procedures, reducing rates of serious postoperative adverse events (Stein at al, 2011).

The microscope light-induced retinal toxicity after cataract surgery has been described in several reports even in short procedures; however, this potential toxicity has not been evaluated by objective criteria (Kleinmann et al, 2002). Indeed, this retinal phototoxicity would be increased for patients with mild macular diseases such as early stages of AMD (Aged Macular Degeneration) (ie: drusen) which are frequently associated in elderly patients with cataract. A systematic literature review of 129 664 individuals estimated the prevalence of early macular degeneration to 8.01% in population aged 45 to 85 years (Wong et al, 2014).

2.2. STUDY HYPOTHESIS AND EXPECTED RESULTS

A three-dimensional (3D) heads-up system (NGenuity®, Alcon, Fort Worth, TX) was recently introduced for both vitreoretinal and cataract surgery, radically improving the quality of surgical visualization. This system allows performing cataract surgery at a lower fraction of light intensity reducing patient's photophobia complaints during surgery and probably preventing potential phototoxic macular injury in cataract surgery.

In a recent pilot study, the Ngenuity system was reported to decrease significantly the operative light intensity and to contribute to a faster visual recovery relative to traditional microscopes in series of 35 patients (51 eyes) who underwent cataract surgery (Rosenberg at al, 2021).

The aim of the study will be to assess the potential functional macular effects by focal and multifocal ERG (electroretinogram) after cataract surgery with NGenuity by comparison to

Standard Operating Microscope (SOM). This study would particularly address eyes at risk for macular toxicity like patients with:

-early AMD: medium-sized drusen (between 63µm and 125µm in diameter)

-intermediate AMD with large drusen (>125 µm) or pigmentary abnormalities in the retina with at least medium-sized drusen (Ferris et al, 2013).

The study hypothesis is using Ngenuity could reduced the light toxicity in eyes of patients with early AMD undergoing cataract surgery compare to traditional microscope.

2.3. STUDY DESIGN RATIONALE

In order to compare the retinal toxicity of patients who underwent cataract surgery with two light exposures, the study will be prospective, randomized in two-parallel group. The clinical investigation will be controlled since the retinal toxicity of patients who underwent surgery with low-intensity exposure (NGenuity) will be compare to those with SOM. The patient will not be able to know which one is used during the surgery, so the patient will stay masked during all the clinical investigation. The surgeon using the device will not be able to be masked. Even if cataract surgery is one of the most commun surgery in ophthalmology, Ngenuity is available only in few centers in France. The center having a strong cataract surgery activity, the clinical investigation will be in a single-center.

2.4. BENEFIT / RISK BALANCE

There is no expected risks by using of the Ngenuity or the SOM. The main risks for patients enrolled in the clinical investigation are related to surgery. Complications after cataract surgery are uncommon, and most of them can be treated successfully. These risks include inflammation, infection, bleeding, swelling, drooping eyelid, posterior capsular bag rupture glaucoma. These risks are identical whatever the arm.

To measure the phototoxicity in the retina, fERG and mfERG, both non-invasive examination will be performed. No pain or no risk is associated to these exams. Nevertheless, ERG requires eye dilation. One drop of Mydriaticum is enough for pupil dilatation and usually required for eye examination during ophthalmology visits. The instillation of Mydriaticum in the study eye will be required specifically for the clinical investigation only at one visit of the follow-up (the day after the surgery), the instillation for the other visits is required by the usual practice. The multifocal ERG requires the instillation of one drop of oxybuprocaïne before the DLT electrode positioning, generating stinging in the eye during few seconds. Oxybuprocaïne is a topical anesthetic routinely used in ophthalmology’s pratice.

The main benefit for patients is for them who are in Ngenuity arm, to have a potential less retinal toxicity due to a low light exposure.

The benefit/risk balance is favorable.

2.5. EXPECTED BENEFITS

Although NGenuity 3D visualization system exist since many years, there is still a lack of objective data showing less retinal phototoxicity with NGenuity by comparison to SOM (standard operating microscope) after cataract surgery. The results of the study (if primary endpoint is achieved) will benefit to the patient, and particularly to patient suffering from early AMD because it will demonstrate that a less phototoxic procedure does exist with less intense light during the cataract surgical procedure.

3. Study objectives

3.1. PRIMARY OBJECTIVE

To assess the change from baseline of retinal response amplitude in voltage in patients one hour after cataract surgery performed with NGenuity compared to standard light condition with SOM.

3.2. SECONDARY OBJECTIVES

The secondary objectives are to assess the change from baseline in patients both arms of:

- amplitude of the response in voltage in patients before surgery, one day and one month after cataract surgery
- latency of response in time in patients before surgery, one hour, one day and one month after cataract surgery
- total photoreceptors response in the macula before surgery, one day and one month after cataract surgery
- visual acuity before surgery, one hour, one day and one month after cataract surgery

4. Endpoints

4.1. PRIMARY ENDPOINT

The primary endpoint is the change from baseline (V1) of photopic b-wave amplitude (µV) measured at V2 (one hour after the cataract surgery) by fERG.

4.2. SECONDARY ENDPOINTS

The secondary endpoints are the change from baseline (V1) of:

- Photopic b-wave amplitude (µV) measured at V3 (post-operative day) and V4 (1 month after surgery) by fERG.
- fERG implicit time (msec) measured at V2; V3 and V4 (another parameter to evaluate cone response)
- Multifocal ERG amplitude (µV) measured at V3 and V4 allowing distinct areas localization of potential damage in the macula. This parameter could not be evaluated at V2 (one hour after the end of surgery) because it requires a good visual acuity
- Visual acuity measured at V2; V3 and V4 (10 scale)

5. Design of clinical investigation

5.1. OVERALL DESIGN

Prospective, randomized, 2-arm controlled, parallel, single-masked, single-center study.

5.2. MEASURES TO MINIMIZE BIAIS

5.2.1. RANDOMIZATION

In order to have 2 strictly comparable groups, the procedure (Ngenuity or SOM) will be randomized for patients requiring cataract surgery with a ratio 1:1. The randomization takes place after checking all eligibility criteria. The randomization list established by the data manager before the start of the clinical investigation will be included in the electronic case report form (e-CRF).

The randomization will be stratified by the cataract grade severity of patients before surgery following matched nuclear cataract (NUC) criteria.

In the WHO/PBD classification system, NUC is defined only in terms of the opacification of the nucleus. The WHO/PBD NUC standards represent increasing opacification from standards 1 to 3 (Thylefors et al, 2002). The physician assigns a severity grade by comparing the degree of opacification in the slit lamp appearance of the grading region with the standard photographs. Grades are assigned as following:

Please note*: The photographs of NUC 1–3 are the* ***standards****; the definitions given below are only to facilitate interpretation of the characteristics for each grade.*

NUC standard 1

In the nuclear zone, the anterior and posterior embryonal nuclei are distinctly more opalescent (more visible) than normally seen, but the central clear zone is still easily distinguishable in its entirety.

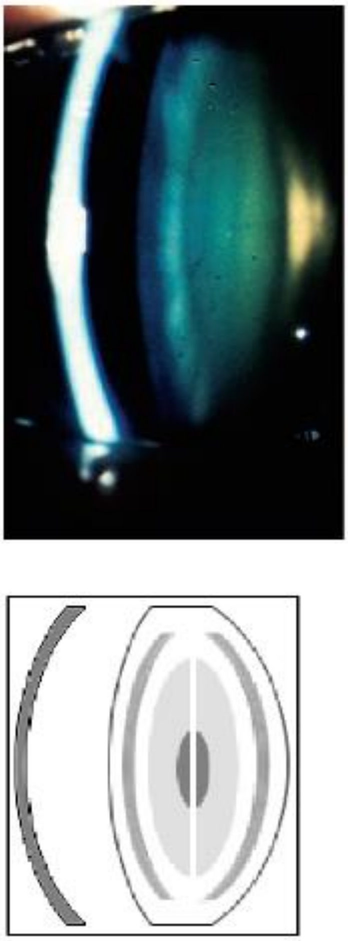

Fig. 2. WHO/PBD NUC standard 1 showing **significant** nuclear cataract formation.

NUC standard 2

The nuclear zone is more uniformly opaque and the central clear zone between the anterior and posterior nuclei is not clearly visible. The posterior part of the zone is often more opaque.

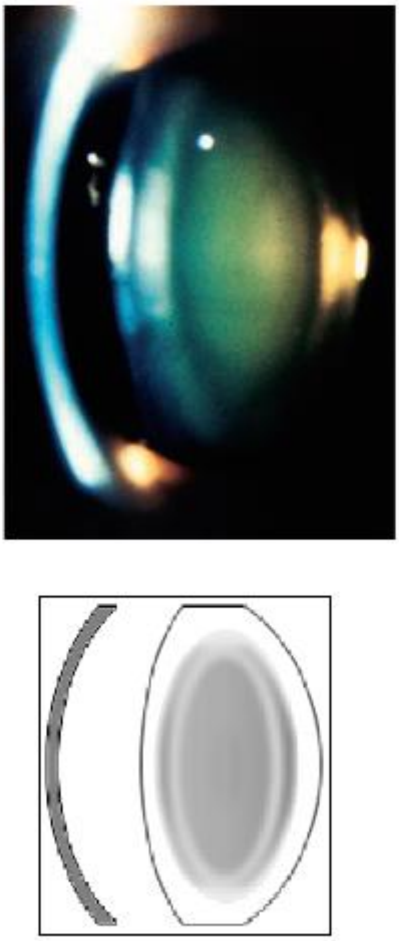

Fig. 3. WHO/PBD NUC standard 2 showing **moderately advanced** nuclear cataract formation.

NUC standard 3

The nuclear zone is densely opaque with more or less uniform nuclear opacity extending to the edge of the nuclear zone; nuclear features are only partially visible, if at all.

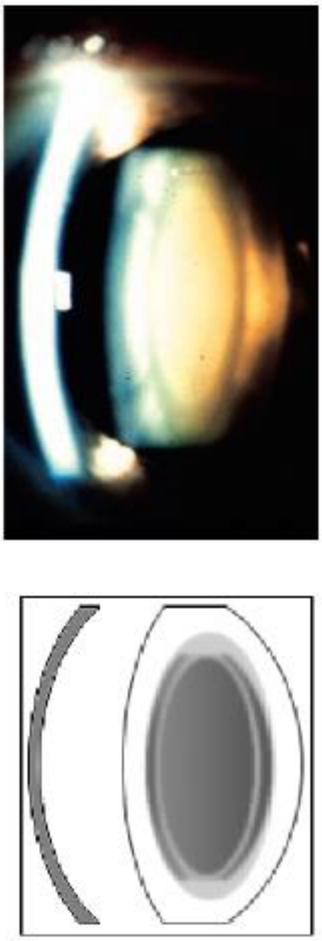

Fig. 4. WHO/PBD NUC standard 3 showing **very advanced** nuclear cataract formation.

5.2.2. Masking procedure

5.2.2.1. Single-masked

The procedure will be masked for the patient undergoing cataract surgery during all the study. It is not necessary that the surgeon was masked, since the main endpoint of the clinical investigation is independently measured by the focal ERG.

5.2.2.2. Unmasking

During the study

The unmasking procedure have to stay ultimate procedure in the aim to guarantee the safety of the patient enrolled in the study or third party. In this study, only the patient is masked, so the investigator will know the arm to guarentee his safety.

If the patient or third party estimates the need to know the procedure following an adverse event occurring between the surgery and the 1-month post-operative visit, he has to contact the investigator. The procedure should be unmasked in agreement with the sponsor.

All unmasking procedure has to be notified in the e-CRF and the event leading to unmasking procedure should be notified to the sponsor via the e-CRF.

At the end of the study

In all other cases, the patient will know the procedure arm at the end of study under request to the investigator.

6. STUDY POPULATION

6.1. INCLUSION CRITERIA

- Patient undergoing cataract surgery and presenting signs of early AMD with medium-sized drusen (between 63 µm and 125 µm in diameter) or intermediate AMD with large drusen (>125 µm) or pigmentary abnormalities with at least medium sized drusen in the study eye.
- Patient affiliated to health care system
- Patient with a signed informed consent

6.2. EXCLUSION CRITERIA

- Children under 18 years old
- AMD defined by geographic atrophy or pigment epithelium detachment, subretinal hemorrhage or visible subretinal new vessel, or subretinal fibrous scars in the study eye
- Non-age-related cataract
- History of previous ocular surgery, anterior segment (corneal, anterior chamber, sulcus) or posterior segment (uveal, vitreo-retinal) pathology including retinal vascular occlusive disease, retinal detachment or peripheral retinal laser photocoagulation, neovascular AMD, glaucoma in the study eye
- Any current anterior or posterior segment inflammation of any etiology, and/or history of any disease producing an intraocular inflammatory reaction.

- Patient with conditions that increase the risk of zonular rupture during cataract extraction procedure that may affect the postoperative centration or tilt of the IOL
- Patient treated by plaquenil
- Patient already enrolled in the same study for one eye
- Patient already enrolled in clinical trial or with exclusion period in progress
- Patient under guardianship, curatorship or other legal protection, deprived of liberty by judicial or administrative decision
- Pregnant or breastfeeding women
- Patient hospitalized without consent

6.3. ENROLLMENT MODALITIES

Patients requiring cataract surgery and presenting signs of early AMD or intermediate AMD in the study eye will be enrolled during consultation of ophthalmology with study investigator in the Hospital Guillaume de Varye. During the visit, the investigator will present the main objectives of the study and propose to the patient to participate. A written information notice with consent form will be given to the patient. Patients will come back for pre-operative visit to estimate the size of IOL (intraocular lens) usually one week before the surgery. Patients who agreed to participate in the study will sign the informed consent form.

On the hundred cataract surgeries performed each month in the hospital, enrollment of 10 patients per month is expected. The enrollment duration of 6 months will be sufficient to reach the target of 42 patients required for the study.

7. STUDY DEVICE

7.1. STUDY DEVICE: NGENUITY

The cataract surgery performed by the surgeon after local anesthesia under microscope as usual practice. During the surgery, the surgeon will use the NGenuity 3D system, allowing a reduced light intensity to 15% (in average). The surgeon is free to adjust the light intensity (increase or decrease) to be in the optimal condition for the surgery. The light exposure during surgery will be measured by luxmeter.

7.2. COMPARATOR DEVICE: SOM

The cataract surgery will be performed under microscope following the usual practice of the surgeon without the NGenuity 3D system, leading to light intensity of 60%, similar to one provided by a Standard Operating Microscope.

7.3. MEDICAL DEVICE AVAILABILITY

The same microscope (ZEISS) will be used for the patients in both arms.

The 3D digital visualization system (NGENUITY®, CE mark) from Alcon (Forth Worth, TX) consists of a 3D stereoscopic, high-definition digital video camera and workstation to provide magnified stereoscopic images of objects during micro-surgery. NGENUITY® is already available on site since 5 years. All patients enrolled in the study will undergo cataract surgery in the same conditions.

8. Treatment and procedure associated

8.1. PROCEDURE ASSOCIATED: ERG

The ERG (electroretinogram) is standard ophthalmic exam to measure the electrical activity of retina (cones/rods) and inner layer (bipolar cells) in response to light exposure. ERG will be performed accordingly to ISCEV (Internal Society for Clinical Electrophysiology of Vision) standard protocol including: pupil dilatation and 10 min light adaptation (Robson et al, 2022). A ganzfeld (whole field) stimulator delivers diffuse flashes that evenly illuminate the maximal area of retina. In the study, only cone response will be assessed with: flash strength of 3 phot cd s m^{-2}, superimposed on a light-adaptating background (luminance 30 cd m-2) as single flashes and at a frequency of 30 Hz will be used. The cone response will be registered using 3 skin electrodes for the focal ERG: one on the forehead (neutral electrode), outer canthus (reference electrode) and one on external lower eyelid of the study eye (active electrode). For the multifocal ERG, the signal registered by active skin electrode is too low. So for multifocal ERG, a DLT electrode (Dawson Litzkow Trick) as active electrode will be placed at the inferior conjunctival cul-de-sac of the study eye, as usually performed in clinical trial on ERG.

The single flash has:
-a-wave dominated by the activity of cone with Off-bipolar cells
-b-wave dominated by a combination of On- and Off- bipolar cells activity, with contributions mediated by cone mechanisms.
The implicit time is defined by the time needed for the electrical response to reach maximal amplitude (see figure below).

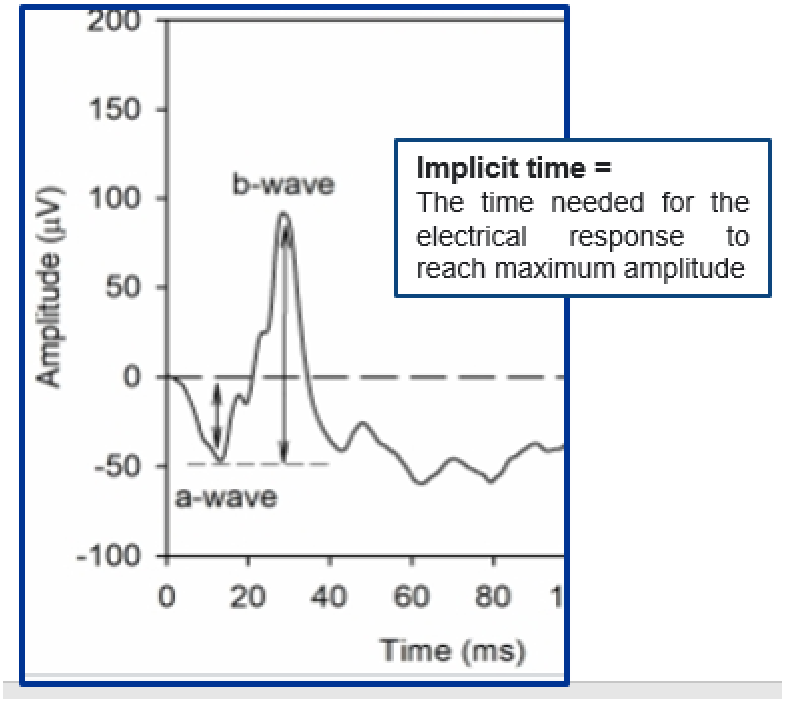

The b-wave amplitude and implicit time are common clinical index of the functional integrity of the cone system.

8.2. AUTHORIZED CONCOMITANT MEDICATIONS

All treatments required for the medical care of patient are authorized.

8.3. PROHIBITED CONCOMITANT MEDICATIONS/PROCEDURES

No treatment or procedure is prohibited during the clinical investigation. If additional procedures are required due to AE that jeopardize the main objective of the study, it could be a reason for early study termination

9. Study procedures

9.1. STUDY DURATION

- Inclusion duration : 6 months
- Individual duration : max 2 months
- Total research duration: 8 months

9.2. SCHEDULED OF ASSESSMENT

	Visit 1 (V1)	Visit 2 (V2)	Visit 3 (V3)	Visit 4 (V4)
	Pre-operative	Surgery	Day after surgery	1 month Postoperative visit
	D0 – 1M to D0 – 7 days	D0	D0 + 1 day	D0+ 1M +/- 5 days
Informed consent	✓			
Eligibility criteria	✓			
Demography (1)	✓			
Medical/ Surgical history	✓			
Visual Acuity	✓	✓	✓	✓
Clinical examination (2)	✓		✓	✓
Focal ERG (R)	✓	✓	✓	✓
Multifocal ERG (R)	✓		✓	✓
Randomization (R)	✓			
Cataract surgery		✓		
CDE (3)		✓		
Light exposure measurement (4)		✓		
Adverse events		✓	✓	✓

(1) Age, gender
(2) Clinical examination : slit lamp examination, fundus examination, OCT
(3) CDE: Cumulated Dissipated Energy measurement
(4) Light of microscope will be measured by luxmeter

(R) procedure required by the clinical investigation
(*) Time unit : M (month) D (day)

9.3. SCREENING VISIT

The First visit is the screening visit. During this visit, the surgeon will meet the patient between 7 days and until 1 month before the scheduled surgery. The Visit 1 (V1) will include:

- Checking eligibility criteria
- Signature of the informed consent
- Demography (age, gender)
- Medical and surgical history
- Visual acuity (snellen/log mar) with correction
- Clinical examination : slit lamp examination, fundus examination, OCT
- Focal ERG on study eye
- Multifocal ERG one study eye
- Randomization performed by surgeon at the closest date before the surgery.

9.4. VISIT 2

The visit 2 (V2) is the day of cataract surgery. The surgeon will perform the study procedure following the arm allocated by randomization. The light exposure during surery will be measured by a luxmeter. Cumulated Dissipated Energy measurement will be extracted from phacoemulsifier apparatus at the end of the surgery.

At the exit of operating room, the patient will stay in the post-operative recovery room where the assessments will be performed one hour after surgery:

- Post-operative visual acuity (snellen/log Mar) without correction
- Post-operative Focal ERG on study eye
- Adverse events.

9.5. FOLLOW-UP VISIT

The first follow-up visit is the day after the surgery. The patient will return to the Hospital for post-operative visit. The visit 3 (V3) will include:

- Visual acuity (snellen/log mar) with correction
- Clinical examination : slit lamp examination
- Focal ERG on study eye
- Multifocal ERG on study eye
- Adverse events.

9.6. LAST VISIT

The last visit is the second follow-up visit occurring 1 month after the surgery. The patient will return to the Hospital for post-operative visit. The Visit 4 (V4) will include:

- Visual acuity (snellen/log mar)
- Clinical examination : slit lamp examination, fundus examination, OCT
- Focal ERG on study eye

- Multifocal ERG on study eye
- Adverse events.

There is no exclusion period in this clinical investigation.

9.7. DISCONTINUATION FROM THE STUDY

9.7.1. WITHDRAWAL

Patients have the right to withdraw voluntarily from the study, especially for consent withdrawal, at any time for any reason. In addition, the investigator has the right to withdraw a patient from the study at any time.

If a patient withdraws prematurely from the study, the reason for patient withdrawal must be documented in the case report form (CRF). The CRF has to be filled-up until the exit of the clinical investigation.

After clinical investigation withdrawal, patients will be treated and followed up as per local practices.

In the case of patients lost to follow-up, the investigator will make all attempts to contact the patient and will notify in the patient’s medical records.

9.7.2. EARLY TERMINATION

The investigator would interrupt temporarily or terminate the participation of patients in the clinical investigation for any reason which deserve the best interests of the patient, especially in case of SAE. The patients will not be follow-up anymore as described in the clinical investigation but will bel treated and followed up as per local practices.

If there is any complication during the cataract surgery, this will be a reason to terminate the clinical investigation earlier.

For any reasons of early termination of patient, the investigator has to notify it with the most of details as possible. The CRF will be filled-up until the exit of the clinical investigation.

The sponsor has the right to terminate the study at any time. Reasons for terminating the study may include, but are not limited to, the following:

- Patient enrollment is unsatisfactory
- Potential health hazard to patients

In case of premature study discontinuation, the sponsor has to declare the end of study with a justification about the premature termination.

9.7.3. ELIGIBILITY CRITERIA VIOLATIONS

A patient included in the clinical investigation who not validated all eligibility criteria has to be discussed. He should be follow-up as scheduled in the clinical investigation until the sponsor made a decision with the lead investigator’s agreement.

9.7.4. PROTOCOL DEVIATIONS

Any deviations of the clinical investigation has to be notified by the investigator. Only withdrawals lead to the end of the follow-up. Even in case of clinical investigation’s deviations, the follow-up of patient will be proceed until the completion of clinical investigation.

9.8. CONSTRAINS RELATED TO THE CLINICAL INVESTIGATION

A patient recruited in the clinical investigation is not authorized to participate to another clinical research during all the clinical investigation duration. There is no exclusion period in this clinical investigation. The patient will not receive any compensation for his clinical investigation's participation.

10. Adverses Events

10.1. DÉFINITIONS

According with article 2 of MDR (EU) 2017/745, the following definitions applied:

Adverse Event (definition 57)
Adverse event (AE) means any untoward medical occurrence, unintended disease or injury or any untoward clinical signs, including an abnormal laboratory finding, in patients, users or other persons, in the context of a clinical investigation, whether or not related to the investigational device.

User means any healthcare professional or lay person who uses a device (definition 37).
Lay person means an individual who does not have formal education in a relevant field of healthcare or medical discipline (definition38).

Serious Adverse Event (definition 58)
Serious adverse event (SAE) means any adverse event that led to any of the following:
- death,
- serious deterioration in the health of the patient, that resulted in any of the following:
  - life-threatening illness or injury,
  - permanent impairment of a body structure or a body function
  - hospitalisation or prolongation of patient hospitalisation
  - medical or surgical intervention to prevent life-threatening illness or injury or permanent impairment to a body structure or a body function
  - chronic disease
- foetal distress, foetal death or a congenital physical or mental impairment or birth defect

Device deficiency (definition 59)
Device deficiency means any inadequacy in the identity, quality, durability, reliability, safety or performance of an investigational device, including malfunction, use errors or inadequacy in information supplied by the manufacturer.

As reminder, the definitions applied in the context of materiovigilance are similar as the post-market surveillance of medical devices:
Incident (definition 64) means any malfunction or deterioration in the characteristics or performance of a device made available on the market, including use-error due to ergonomic features, as well as any inadequacy in the information supplied by the manufacturer and any undesirable side-effect.

Serious incident (definition 65) means any incident that directly or indirectly led, might have led or might lead to any of the following:

- the death of a patient, user or other person,
- the temporary or permanent serious deterioration of a patient's, user's or other person's state of health,
- a serious public health threat (66) defined as an event which could result in imminent risk of death, serious deterioration in a person's state of health, or serious illness, that may require prompt remedial action, and that may cause significant morbidity or mortality in humans, or that is unusual or unexpected for the given place and time;

10.2. EVALUATION OF RELATIONSHIP

The sponsor and the investigator shall distinguish the SAE that has a causal relationship with the investigational device or the comparator, or with the investigational procedure.

It is required to discriminate:

- **Additional procedures (in addition to the current use of the medical device):**

In order to classify the clinical investigations related to medical device with CE mark, used in the limits of the use allowed in CE marking, additional procedures means all procedures required in the clinical investigation and which are additional to the usual use of the medical device included in CE marking.

- **Investigational procedure (prior the SAE):**

In order to notify accordingly to article 80 of MDR, an SAE occurring during the clinical investigation, investigational procedure prior the event means all activities related to use of medical device, under clinical investigation, required by the protocol and initiated prior the occurrence of the AE or concomitant to the AE, that have a causal relationship suspected with the event.

The sponsor and the investigator shall defined the causal relationship:
- **unrelated**: the causal relationship with the medical device, the comparator or the procedures is excluded
- **possibly related**: the causal relationship with the medical device, the comparator or the procedures is low but could not be totally excluded. Others reasons are possible as disease, underlying or concomitant clinical conditions, other treatment or drug effect. In the case where the evaluation of causal relationship is not possible or if there is no information about it, the event shall be defined as possibly related.
- **probably related**: the causal relationship with the medical device, the comparator or the procedures seems relevant and/or the event is not explained by another reason.
- **related**: the SAE is related to use of the medical device, the comparator or the procedures.

10.3. EVALUATION OF THE INTENSITY

The investigator has to grade the intensity of the AE as following:

- Grade 1 (light): usually temporary AE without any interference on usual activities
- Grade 2 (moderate): AE enough bothering with interference on usual activities
- Grade 3 (severe): AE modifying substantially the usual activities or disabling, or life threatening

10.4. NOTIFICATION BY THE INVESTIGATOR TO THE MATERIOVIGILANCE

The investigator has to notify any AE/device deficiency/incident likely **related exclusively to the medical device** to the local materiovigilance representing immediately after being aware

10.5. NOTIFICATION BY THE INVESTIGATOR TO THE SPONSOR

The investigator has to notify the sponsor **immediately** and not more than 3 days after being aware, the following events defined in the section 11.1 accordingly to article 80 of the MDR:

a) Any AE of a type identified in the clinical investigation plan as being critical to the evaluation of the results of that clinical investigation

b) Any SAE

c) Any device deficiency that might have led to a SAE if appropriate action had not been taken, intervention had not occurred, or circumstances had been less fortunate

d) Any new findings related to any event referred to in points a) to c)

All these events should be recorded on the e-CRF from the time the patient signed content through last patient visit. The entry in the e-CRF will generate an automatic sending of e-mail to the sponsor for notification.

10.6. NOTIFICATION BY THE SPONSOR TO AUTORITIES

The sponsor or its representative (or legal representative) to which the sponsor delegated the notification, shall report without delay to the authorities accordingly to article 80 of MDR:

a) Any serious adverse event that has **a causal relationship with the investigational device**, the comparator or the investigation procedure or where such causal relationship is **reasonably possible**

b) Any device deficiency that might have led to a serious adverse event if appropriate action had not been taken, intervention had not occurred, or circumstances had been less fortunate

c) Any new findings in relation to any event referred to in points (a) and (b)

10.7. TIMELINE AND REPORTING MODALITIES BY THE SPONSOR TO AUTORITIES

The period for reporting shall take account of the severity of the event. Where necessary to ensure timely reporting, the sponsor may submit an initial report that is incomplete followed up by a complete report.

All events or device deficiency presented in section 11.6 led to death or a life threatening , injury or serious illness, requiring an urgent corrective action for the patients, users or other persons, or all new finding related to these events:

- Immediately and **no more than 2 days** at the time when the sponsor has been aware of the event or new information related to the event requiring notification to authorities.

The others events and device deficiency presented in section 10.6 or all new findings about the events:

- Immediately and **no more than 7 days** at the time when the sponsor has been aware of the event or new information related to the event requiring notification to authorities

Starting from May 26th 2021 and awaiting the delivery of EUDAMED database, SAE and device deficiencies under the clinical investigation presented in section 11.6 has to be declared to:

- Regulatory authorities (ANSM) as described below:
  - Under table format like presented in the investigation summary safety report form (MDCG-2020-10/2) available to:
  https://ec.europa.eu/health/sites/health/files/md_sector/docs/md_mdcg_2020-10-2_guidance_safety_report_form_en.xlsx.
  The table will be updated and sent each time that a new event has to be declared or that a new information related to event already declared has to be notify.
  - By e-mail to: EC.DM-COS@ansm.sante.fr with a title as following: AAAAMMJJ_SAE_N°IDRCB or N°EUDAMED or via EUDAMED as soon as it will be available.
- And, if necessary, in the same time to all authorities of Member States in which the clinical investigation is being conducted.

10.8. CLINICAL INVESTIGATION REPORT

The clinical investigation report will include a summary of SAE, adverse device effects, device deficiencies and any relevant corrective actions as required by the chapter III of MDR.

11. Data management

In accordance with Article 72 of the MDR, all information related to the clinical investigation will be recorded, processed, handled and stored by the sponsor or investigator, as the case may be, in such a way that it can be accurately reported, interpreted and verified, while preserving the confidentiality of the recordings and of the personal data of the patients, in accordance with the applicable legislation on personal data protection.

All data required for the clinical investigation will be from medical report of patients, completed by the investigator during the visits. The data will be recorded on eletronic case report form (e-CRF). The tool verified and secure, hosted on authorized health data server, will be validated by the sponsor and the investigator.

Only essential data required for the clinical investigation will be collected by the investigators (or other authorized persons) without showing any name address. Each patient will have unique identification number, containing two digits for center number followed by 2 digits for the recruitement order in the center.

Each investigator or any other authorized persons entered collected data in the e-CRF as clinical investigational progresses. Each users will have its own username and password to connect, enter or correct data in the e-CRF. Each modification of data will be recorded by an audit-trail. Data entered must be accurate and complete, and will be checked and validated accordingly to the sponsor's procedures. Automatic consistency checks, in addition to other checks, will ensure the consistency of the data and the detection of aberrant, missing or erroneous data. If necessary, requests will be sent to the center for correction. Until no further requests pending, the principal investigator will validate the data by signing electronically the e-CRF of each patient enrolled.

12. Statistic methods

12.1. SAMPLE SIZE CALCULATION

The patients to include in this clinical study, representing 10% of patients, will be recruited among the active patient list of about 600 patients during the 6 months enrollment period.

Under the following hypotheses:

- Superiority study
- Main evaluation criterion = Photopic b-wave amplitude
- Continuous parameter
- 2 parallel groups
- Alpha = 5%
- Beta = 10% (power = 90%)
- Two-sided situation
- Standard deviation (SD) = 3,75 (Mean SD between 2,9 and 4,6, from Larsson et al, 2001)
- Expected difference from baseline on photopic b-wave for :
  - studied group = 20% (from Larsson et al, 2001)
  - standard group = 0%
- Expected difference between the two groups = 20%
- R software used for sample size calculation
- As the study will include patients according to their cataract grade (3 strata), the number of patients to include in each stratum must be a multiple of 6.

If we consider a **15%** drop out (loss of FU, ERG impossible to realize at each visit, etc …): a total of **42 patients: 21 patients/arm** would be included.

12.2. POPULATION ANALYSIS

The follwing analysis populations will be defined:

- Population of included patients (PI): All patients included in the study with at least one available data.
- Per-protocol Population (PP): Patients included in the PI population without major protocol deviations.

The major protocol deviations will be listed in the Data Management Report (DMP).

12.3. STATISTIC ANALYSIS

12.3.1. OVERALL CONSIDERATIONS

12.3.1.1. STATISTIC SOFTWARE

A full description of the statistical methods planned for this study will be provided in the Statistical Analysis Plan (SAP). Statistical analysis will be performed using R software (at least version 4.2.0) or equivalent after the database freeze.

12.3.1.2. MISSING DATA MANAGEMENT

The missing data will not be replaced but the number of missing data will be included in the descriptive statistics.

12.3.1.3. DESCRIPTIVE STATISTICS

All study parameters (population characteristics, primary and secondary evaluation criteria) will be described according the type of parameter:

- Quantitative data: number of missing data, sample size, mean, standard deviation, median, first and third quartiles, minimum and maximum
- Qualitative data: number of missing data, sample size, percentage of each item and in total. The percentage will be calculated regardless of missing data.

12.3.1.4. STATISTICAL TESTS

The following statistical tests will be performed according the type of parameters:

- Quantitative data: Student's test or Wilcoxon-Mann-Whitney' test according the data distribution
- Qualitative data: X^2 test or Fisher's exact test if at least one sample size in contingence table is lower than 5.

All statistical tests will be perfomed in two-side situation and with a significance level of 5%.
The 95% confiance intervals (CI) à 95% can be calculated if necessery.

12.3.2. DESCRIPTION OF POPULATION

The description of the PI and PP populations by technic group and stratification factor (NUC) will carry on the following parameters measured in the randomization visit:

- Demography (age, gender)
- At least one medical and surgical history
- Visual acuity (snellen/log mar)

- Slit lamp examination, fundus examination, OCT
- Per-operative Focal ERG
- Per-operative Multifocal ERG

and the following parameters measured in the surgery visit:

- Post-operative visual acuity (Snellen/log Mar)
- Post-operative Focal ERG.

The comparison of 2 technic groups will to verify the homogeneity of the study population.

12.3.3. Analysis of the main objective base on principal endpoint

The main study objective is to assess retinal phototoxicity in patients immediately after cataract surgery performed with NGenuity compared to standard light condition with SOM. The photopic b-wave (µV) describing the retinal phototoxicity is evaluated at V2 after the cataract surgery.
This parameter is a quantitative data.
On the PP population, the statistical methodology will be set up to answer this question:

- Descriptive statistics at V1 and V2 by technic groups and in total
- Description statistics of the difference from baseline (V1) by technic groups and in total
- Model of Analysis of covariance (ANCOVA) including the following factors:
  - Fixed factor : technic group (Ngenuity, SOM),
  - Repeated factor: time (V1 and V2),
  - Interaction time*group,
  - Covariate: baseline value (V1).

  The validity conditions of ANCOVA will be verify before the analysis. In case of at one condition will not be respected, a data transformation or a model of generalized estimated equations (GEE) including the same factors, interaction and covariate will be performed.
- Graph of change parameter : time in x-axis and mean and CI b-wave in y-axis

The p value of interaction will give the answer of main objective.

12.3.4. Analysis of secondary objectives based on secondary endpoints

The secondary goals are to describe retinal phototoxicity with an objective methods in low light condition (NGenuity) by comparison to standard light condition with SOM after cataract surgery for eyes with presence of drusen in the retina (early AMD), which are more likely to experience a reduction in ERG retinal signalling, with the following outcomes:

12.3.4.1. Retinal phototoxicity from V1 to V4

The retinal photoxicity is described by the photopic b-wave (µV) measured by fERG at V3 and V4 after the cataract surgery.
On the PI population, the statistical methodology will be set up to answer this question:

- Descriptive statistics at V3 and V4 by technic groups and in total
- Description statistics of the difference from baseline (V1) at V3 et V4 by technic groups and in total
- Model of Analysis of covariance including the following factors:
  - Fixed factor: technic group (Ngenuity, SOMl),
  - Repeated factor: time (V1, V2, V3 and V4),

  - Interaction time*group,
  - Covariate: baseline value (V1).

  The validity conditions of ANCOVA will be verify before the analysis. In case of at one condition will not be respected, a data transformation or a model of generalized estimated equations (GEE) including the same factors, interaction and covariate will be performed.
- Graph of change parameter : time in x-axis and mean and CI b-wave amplitude in y-axis

The p value of interaction will give the answer of this secondary objective.

12.3.4.2. Cone response on from V1 to V4

The cone response is also described by the fERG implicit time (msec) measured at V2, V3 and V4 after the cataract surgery.

On the PI population, the statistical methodology will be set up to answer this question:

- Descriptive statistics at V1, V2, V3 and V4 by technic groups and in total
- Description statistics of the difference from baseline (V1) at V2, V3 et V4 by technic groups and in total
- Model of Analysis of covariance including the following factors:
  - Fixed factor: technic group (Ngenuity, SOM),
  - Repeated factor: time (V1, V2, V3 and V4),
  - Interaction time*group,
  - Covariate: baseline value (V1).

  The validity conditions of ANCOVA will be verify before the analysis. In case of at one condition will not be respected, a data transformation or a model of generalized estimated equations (GEE) including the same factors, interaction and covariate will be performed.
- Graph of change parameter : time in x-axis and mean and CI b-wave amplitude in y-axis

The p value of interaction will give the answer of this secondary objective.

12.3.4.3. Other damage from V1 to V4

The distinct areas localization of potential damage in the macula is described by the multifocal ERG amplitude (µV) measured at V3 and V4.

This parameter could not be evaluated at V2 (15 min after the end of surgery) because it requires a good visual acuity

On the PI population, the statistical methodology will be set up to answer this question:

- Descriptive statistics at V1, V3 and V4 by technic groups and in total
- Description statistics of the difference from baseline (V1) at V3 et V4 by technic groups and in total
- Model of Analysis of covariance including the following factors:
  - Fixed factor: technic group (Ngenuity, SOMl),
  - Repeated factor: time (V1, V3 and V4),
  - Interaction time*group,
  - Covariate: baseline value (V1).

The validity conditions of ANCOVA will be verify before the analysis. In case of at one condition will not be respected, a data transformation or a model of generalized estimated equations (GEE) including the same factors, interaction and covarite will be performed.

- Graph of change parameter : time in x-axis and mean and CI multifocal ERG amplitude in y-axis

The p value of interaction will give the answer of this secondary objective.

12.3.4.4. Visual acuity From V1 to V4

The Visual acuity measured at V2, V3 and V4 after the cataract surgery is also analysed.
The same statistical methodology to be use for the fERG implicit time will be performed.

12.3.5. Interim analysis

No interim statistical statistical analysis is planned.

13. Clinical investigation oversight

13.1. COMMITTEE

Regarding the elements detailed in the clinical investigation section 3.4, a specific research committee is not deemed necessary for the study.

13.2. QUALITY CONTROL

The sponsor will appoint a monitor that is independent from the investigational site to ensure that the investigation is conducted in accordance with the clinical investigation, the principles of good clinical practice and the MDR as required in the annex XV, chapter III point 4. During these visits, the monitoring will be performed as described in the monitoring plan and will cover:

- Signed consent by each patient enrolled in the clinical investigation,
- Respect of procedures described in the clinical investigation
- Medical device tracking if necessary
- AE and SAE occurrence and notification to materiovigilance and/or to the sponsor depending of the type of AE
- Documents which should be present in the clinical investigation master file
- Quality of data recorded in the e-CRF: accuracy, missing data, consistency with original data sources (clinical report, hospitals record, laboratory results…..)

A report will be written after each visit.

13.3. AUDIT AND INSPECTION

An audit can be performed during all the clinical investigation by persons mandated by the sponsor independently of investigor's site. The aim is to check the quality of the clinical investigation, the validity of the results and the respect of the current regulations.
The investigator agree to comply with the sponsor's requirements for an audit and the competent authorities for inspection.

The audit will be performed at any stage of the clinical investigation, from the protocol writing to publication of results or archiving of data used or produced during the clinical investigation.

14. Access to data and source documents

14.1. ACCESS TO DATA

The sponsor must get the authorization of all persons implicated in the clinical investigation in order to be sure that source data, source documents, investigational center, and study reports are directly accessible with the aim of quality control and audit by the sponsor.
To facilitate source data verification, the investigators and institutions must provide the sponsor (or designee) direct access to applicable source documents and reports for trial monitoring and sponsor audits. The center must also allow inspection by applicable health authorities, according to the articles L.1121-3 and R.5121-13 of the french public health code.

14.2. SOURCE DATA

All informations included in original documents or certified copies of documents after verification as being accurate and complete, related to clinical examinations, evaluation or other activities performed during the clinical investigation are source data. Source documents (paper or electronic) are those in which patient data are recorder and documented for the first time.

14.3. CONFIDENTIALITY

In accordance with the Chapter IX Article 109 of European Regulation EU 2017/745, persons having direct access to the source data take all the necessary cautions to ensure the confidentiality relating to medical devices, investigations clinics, to the persons who take part in them and in particular with regard to their identity as well as to the results. These people, like the investigators themselves, are under professional secret.

The sponsor will make sure that each patient enrolled in the clinical investigation is agree to give access to individual data about him and strictly required for the quality control of the clinical investigation.

14.4. PERSONAL DATA PROTECTION

All following requirements agree of the new regulation of the personal data protection: limited access, secure access, identification coding, saving and archiving procedures for the data management and the information of patients with GPDR annex in the information note presenting the aim of the clinical investigation, the type of data collected, the data processing and the behavior of the data. It is included the rights of patients to access, correct, suppress or also be aware of data portability… and to contact a DPO (data protection officer).

15. Ethical and regulatory considerations

15.1. ETHICAL CONSIDERATIONS

The sponsor and the investigator agree to perform the clinical investigation according to the french law n°2012-300 from March 5th 2012 about the investigation on patients, the Good clinical practices (GCP) (I.C.H. version 4 from November 9th 2016 and addition from November 24th 2016) and the declaration of Helsinki (available in full text on http://www.wma.net). The clinical investigation is in line with the international standard ISO 14155:2011 on good clinical practice for clinical investigations of medical devices for human subjects.

The clinical investigation will be performed accordingly to the protocol. Except in emergency that require specific medical care, the investigator will follow all procedures described in this document, especially about the inform consent form and the notification of AE/SAE. The clinical investigation will start only after reception of approval from the ethic committee.

15.2. REGULATORY CLASSIFICATION

This study is qualified as clinical investigation (4.2) on CE marked device, without aim of device conformity assessment, used in its indication and including non-heavy and non-invasive additional procedures according to article 82 of the European regulatory (UE 2017/745) related to medical devices (MDR).

15.3. REGULATORY REQUIREMENTS

15.3.1. Ethic committee

Accordingly, to the MDR, the protocol has been submitted to the French ethic committee and received an approval.

15.3.2. Competent authority

Due to regulatory classification and to be conform to MDR, the clinical investigation has been submitted to competent authority (ANSM, french national agency for the safety of drug and health products) the same day as the ethic committee to get a coordinated validation.

15.3.3. Amendment

If a sponsor intends to introduce modifications to a clinical investigation that are likely to have a substantial impact on the safety, health or rights of the patients or on the robustness or reliability of the clinical data generated by the investigation, it shall notify, within one week, by means of the electronic system referred to in Article 73 the Member State(s) in which the clinical investigation is being or is to be conducted of the reasons for and the nature of those modifications. The sponsor shall include an updated version of the relevant documentation referred to in Chapter II of Annex XV as part of the notification. Changes to the relevant documentation shall be clearly identifiable. Ethic committee approval is required before the implementation of any changes.

All amendments have to be validated by the sponsor, and by all contributors of the clinical investigation, previously to submission to ethic committee and competent authority.

All investigators involved in the clinical investigation have to be aware of all amendments and agreed to be compliant to the amendments.

Each modification about the patient's health care, the benefits, the risks or the constraints led to a modification of the informed consent form, which follow the same process as described in section 17.

15.3.4. CNIL

Data recorded for the clinical investigation will be processed in compliance with:

- Regulation (EU) 2016/679 of the European Parliament and of the Council of 27 April 2016 on the protection of natural persons with regard to the processing of personal data and on the free movement of such data, and repealing Directive 95/46/EC (General Data Protection Regulation)
- Article 66 of the law n°78-17 from January 6th 1978 related to information, files and freedoms modified by the ordinance n°2018-1125 of December 12th 2018 - art. 1.
- Deliberation no. 2018-153 of 3 May 2018 approving a reference methodology relating to the processing of personal data implemented in the context of research in the field of health with collection of the consent of the person concerned (MR -001) and repealing deliberation no. 2016-262 of July 21, 2016.

The sponsor has a declaration of conformity to this reference methodology (MR-001) from CNIL.

15.3.5. Insurance

In accordance with the article 69 of the MDR, the Sponsor has taken out patient liability insurance for all patients who have given their consent to the clinical investigation. This cover is designed for the event that a fatality, physical injury, or damage to health occurs during the clinical investigation's execution. The contract is registered under the number 165261 in Relyens Mutual Insurance, 18 rue Edouard Rochet, 69372 LYON cedex08-France.

15.3.6. Clinical trials

The clincial investigation is registered on the following website: http://clinicaltrials.gov/

16. Rights of patient

16.1. INFORMATION OF PATIENTS

All patients will receive information orally and by written on the notice about the aim of the clinical investigation, the procedures, the duration, the benefits, the potential risks, the constraints due to enrollment in the clinical investigation and the approval of ethics committee (article L.1122-1 of French Public Health Code and article 63 of the RDM). The information form contains all information required to be compliant with the regulation about the personal data protection (GDPR). The investigator will answer all wonderings about the clinical investigation. The investigator will notify the patients about their rights. The investigator will give to all patients an information and consent form.

After full information, the patient will have enough time to think about it and decide if he would like to participate or not to the clinical investigation. The investigator is responsible to get the written informed consent form.

16.2. CONSENT

All patients have to give their written consent to participate to the clinical investigation and to certify their agreement for data collection. The consent form has to be signed before the first clinical examination or act required in the clinical investigation has been performed.

If the patient agree to participate to the clinical investigation, the patient and the investigator have to write their first and last names, dated and signed the consent form in two original copies:
- one consent form is kept by the patient,
- the second consent form is kept by the investigator (even if the patient move out of town during the clinical investigation) in a restricted area.

The investigator has to notify to the patient that he is free to agree or not to participate to the clinical investigation, but also he has the right to withdraw from the clinical investigation at any time for any reasons without any damage or prejudice for his healthcare. The enrollment of the patient in the clinical investigation will be notified in his medical record at the initial visit.

16.3. RIGHTS OF PATIENTS AND DATA ACCESS

In accordance with to the law 2002-303 from March 4th 2002 about the rights of patients and the health system quality, modified by the law n°2016-41 from January 26yh 2016 referred to the health system modernization, the patients have the right to access, during the clinical investigation and at the end, to their medical record.

The medical record of the clinical investigation can be communicated to the patient, like all medical records, accordingly to article L1111-7 of the French Public Health Code.

In accordance with the law about data processing, files and freedoms (article 40, data protection act of January 6th 1978 modified) and the regulation (EU) 2016/679 about protection of personal data, the patient has the following rights:

- To access and to correct data at any time against the principal investigator.
- If the patient wishes to withdraw from the clinical investigation, the data collected before withdraw, can be used like all the others data collected due to public interest of the treatment. So, the sponsor has the right not to follow the rights to delete data already collected and the right to portability. These rights would jeopardize the assessment of the clinical investigation.
- To limit the data processing to the clinical investigation and to refuse using of data for future research.

The patient has the right to refuse to communicate some data under professional secret which could be used and processed in the clinical investigation

The patient will keep the right to access and to correct data against a physician of his choice, but also to refuse using of data. The patient can also contact the principal investigator to assert his rights.

If the patient is not satisfy of the data processing, he can address a complaint to CNIL via the following website: https://www.cnil.fr/fr/plaintes or by mail to: CNIL, 3 Place de Fontenoy - TSA

80715 - 75334 Paris Cedex 07. The patient can also contact the data protection officer (DP0) of the sponsor by e-mail to the following contact: dpo@elsan.care

17. Retention of records

All documents related to the clinical investigation must be retained accordingly to Good Clinical Practices and the current regulation :

- By the principal investigator, during 15 years after the end of the clinical investigation for the documents of the clinical investigation. The medical record has to be retained accordingly to the regulation.
- By the sponsor during 15 years after the end of the clinical investigation

No moving or destroying can be performed without the agreement of the sponsor. At the end of legal time of archiving, the sponsor will be consulted for destroying. All data, documents and reports have to be available for an audit or inspection.

18. Final report

Within one year after the end of the clinicial investigation or within three months following the early termination, the sponsor shall submit a clinical investigation report. This report shall be accompanied by a summary presented in terms that are easily understandable to the intended user. Both the report and summary shall be submitted by the sponsor by means of the electronic system referred to in Article 73 of the MDR.

Where, for scientific reasons, it is not possible to submit the clinical investigation report within one year of the end of the investigation, it shall be submitted as soon as it is available. In such case, the clinical investigation plan referred to in Section 3 of Chapter II of Annex XV shall specify when the results of the clinical investigation are going to be available, together with a justification.

19. Rules related to publication

19.1. SCIENTIFIC COMMUNICATION

The sponsor is the owner of data, analyses and results related to the study, therefore any publication, abstract or oral presentations including results of the clinical investigation must be first submitted to the sponsor for approval. Additionally, all communications, manuscripts or oral presentations must include a section mentioning GCS ELSAN as well as any institution, physician-investigators, methodologists, statisticians and data manager that has contributed to the clinical investigation, including organizations that have provided financial support. International writing and publication rules will be applied (the Uniform requirements for Manuscripts of the ICMJE, April 2010).

The national coordinator will be the main writer of the publication and the first or last author. The national coordinator may however designate another person to (co-) write the publication.

Affiliation of authors with an activity in health care center of ELSAN group should mentioned in the publication : **« ELSAN, Name of the hospital where the clinical investigation has been conducted, City, Country».**

19.2. COMMUNICATION OF RESULTS TO PATIENTS

According to the law n°2002-303 from March 4th 2002, patients are informed, under request, global results of the research.

20. References

Binns AM and Margrain TH. Invest Ophthalmol Vis Sci. 2007;48:2806–2813. Evaluating Retinal Function in Age-Related Maculopathy with the ERG Photostress Test

Brown NA, Hill AR. Cataract: the relation between myopia and cataract morphology. Br J Ophthalmol. 1987 Jun;71(6):405-14. doi: 10.1136/bjo.71.6.405. PMID: 3620419; PMCID: PMC1041188

Ferris FL 3rd, Wilkinson CP, Bird A, Chakravarthy U, Chew E, Csaky K, Sadda SR; Beckman Initiative for Macular Research Classification Committee. Clinical classification of age-related macular degeneration. Ophthalmology. 2013 Apr;120(4):844-51. doi: 10.1016/j.ophtha.2012.10.036. Epub 2013 Jan 16. PMID: 23332590.

Foster A (2000): Vision 2020: the cataract challenge. Community Eye Health 13: 17– 19.

Hood DC. Assessing retinal function with the multifocal technique. Prog Retin Eye Res. 2000 Sep;19(5):607-46

Kleinmann G, Hoffman P, Schechtman E, Pollack A. Microscope-induced retinal phototoxicity in cataract surgery of short duration. Ophthalmology 2002; 109:334–338

Robman, L., Taylor, H. External factors in the development of cataract. Eye 19, 1074–1082 (2005). https://doi.org/10.1038/sj.eye.6701964

Rosenberg ED, Nuzbrokh Y, Sippel KC. J Cataract Refract Surg. 2021 Mar 1;47:291-296. Efficacy of 3D digital visualization in minimizing coaxial illumination and phototoxic potential in cataract surgery: pilot study

Larsson J, Andreasson S. Br J Ophthalmol 2001;85:683–685. Photopic 30 Hz flicker ERG as a predictor for rubeosis in central retinal vein occlusion

Robson AG, Frishman LJ, Grigg J, Hamilton R, Jeffrey BG, Kondo M, Li S, McCulloch DL (2022). ISCEV Standard for full-field clinical electroretinography (2022 update). Doc Ophthalmol 144(1):165–177

Stein JD, Grossman DS, Mundy KM, Sugar A & Sloan FA (2011): Severe adverse events after cataract surgery among medicare beneficiaries. Ophthalmology 118:1716–1723.

Thylefors B, Chylack LT Jr, Konyama K, Sasaki K, Sperduto R, Taylor HR, West S; WHO Cataract Grading Group. A simplified cataract grading system. Ophthalmic Epidemiol. 2002 Apr;9(2):83-95. doi: 10.1076/opep.9.2.83.1523. PMID: 11821974.

Wong WL, Su X, Li X, Cheung CMG, Klein R, Cheng CY,Wong TY. Lancet Glob Health. 2014;2:e106-16. Global prevalence of age-related macular degeneration and disease burden projection for 2020 and 2040: a systematic review and meta-analysis

Budjet: PAYMENT SCHEDULE

The budget agreed with Alcon Laboratoires SAS for the clinical trial amounts to a maximum of €98,600.00 (ninety-eight thousand six hundred euros).

Payments shall be due and transferred to the Sponsor (ELSAN Healthcare Cooperation Group – Groupement de Coopération Sanitaire ELSAN) in accordance with the following milestones:

Payment Milestones:

Contract execution: €10,000
Estimated date: November 2022

Approval by the Ethics Committee (CPP): €22,800
Estimated date: April 2023

First patient inclusion: €21,343
Estimated date: July 2023

50% of patient inclusion achieved: €7,464
Estimated date: October 2023

Last Patient Last Visit (LPLV): €7,464
Estimated date: February 2024

Statistical analysis report: €10,954
Estimated date: May 2024

Two abstracts and one accepted presentation at international congresses: €3,080
Estimated date: September 2024

Final study report and/or manuscript draft: €12,495
Estimated date: October 2024

Open access publication fee: €3,000
Estimated date: December 2024
